# Utilization of Earth’s Internal Heat

**Published:** 1887-03

**Authors:** 


UTILIZATION OF EARTH'S INTERNAL HEAT.—An attempt is about to be made at St. Augustine, Fla., to sink a twelve-inch artesian well to a depth sufficient to obtain water hot enough to heat buildings, pure enough for domestic purposes and with pressure enough to run heavy machinery. The facts upon which this peculiar experiment are based are that water can be found in Florida by boring two hundred and fifty feet. The artesian wells in that State have considerable pressure, and from a depth of six hundred feet send water of warm temperature to a head of forty-five feet when piped. The question of running heavy machinery by artesian well power is by no means purely experimental—it is done in many locations in France already, and the experience of the French shows that the deeper the well the greater the pressure and the higher the temperature. At Grenelle, France, a well sunk to the depth of 1,802 feet and flowing daily 500,000 gallons, has a pressure of sixty pounds to the square inch, just double that of the 600-foot well at Jacksonville, and the water from this Grenelle well is so hot that it is used for heating the hospitals in the vicinity.—*Am. Meteorological Journal.*

---

Woitkewitch has made seventy-one observations of the effects produced by the inhalation of cold air by fever patients. Although no very marked therapeutic results were obtained the experiments show that there is but little if any danger in allowing a fever patient to breathe what he so much craves, cold air. The following facts were learned as the results of the author's study:

1. Inhalations of cold air have but little influence in lowering the temperature of the body. When the temperature is slightly reduced it rises after the inhalation of cold is stopped.

2. The pulse and respiration are reduced in frequency in a marked manner.

3. The general condition of the patient and sleep are improved, but this effect is not permanent.

4. Cold air diminishes the symptoms of bronchial catarrh.

5. Cold air exerts no injurious influence upon the progress of a disease, but on the contrary the writer states that he has found this treatment of undoubted therapeutic value.—*L'Abeille Medicale.*

